# Zinc–Air Hearing Aid Batteries: An Analysis of Functional Performance

**DOI:** 10.3390/audiolres14040056

**Published:** 2024-07-23

**Authors:** James Thomas, Barry Bardsley, Jane Wild, Michael William Owen Penman

**Affiliations:** 1 Adult Audiology & Primary Care Service, Swansea Bay University Health Board, Swansea SA2 8QA, UK; 2 Faculty of Medicine, Health & Life Science, Swansea University, Swansea SA2 8PP, UK; b.bardsley@swansea.ac.uk; 3 Audiology Service, Betsi Cadwaladr University Health Board, Rhyl LL18 5UJ, UK; jane.wild@wales.nhs.uk; 4 Electronics & Software, Team Consulting Ltd., Cambridgeshire CB10 1SX, UK; penman.michael@googlemail.com

**Keywords:** hearing aid battery, disposable battery, zinc–air battery, battery consumption

## Abstract

Background: The aim of this study was to evaluate the performance of three disposable hearing aid battery brands available in Wales. Hearing-impaired individuals who utilise hearing aids rely on the functionality of their devices, which is often contingent upon the quality and longevity of disposable batteries. Materials and Methods: A grey literature review foregrounded the battery standards. The “real-life” use of batteries was supplemented through laboratory testing. Parameters relating to performance quality were used to quantify an overall service life of five PR44- and four PR48-size batteries per manufacturer. Results: The literature review signalled a large gap in hearing aid battery consumption research. All battery brands underperformed compared to their specifications but met IEC standards. Conclusions: Revisions to battery consumption test conditions should reflect new technological features and refine expectations of real-life use. It was possible to statistically identify the best performing hearing aid battery brand.

## 1. Introduction

The composition and performance of zinc–air batteries (ZABs) must meet international standards, and the 1979 Agreement on Technical Barriers to Trade necessitates such surveillance to curb the flow of the global market. This entails testing battery quality according to parameters, ensuring that they meet the specifications required for regulatory inspections, and further, to grant protection to distributors and safety assurance for consumers. While these standards often retain a blanket focus on safety and longevity, there are performance requirements which may not reflect the realistic applications of use, or are yet to be considered and accounted for, perhaps due to the increasing capabilities of modern hearing aids (HAs). The concomitant question of whether the ZAB power supply can follow and meet these demands must be raised. Furthermore, our review of the specifications will focus on the Danalogic Ambio Behind The Ear (BTE) standard hearing aids (SHAs), as these are the predominant model which are distributed to patients in Wales, and supply wireless testing data [1]. The aim of this literature review is to delineate the performance standards which should be detected by laboratory testing.

The term “battery” refers to a portable energy source which is composed of an electrolyte, anode, and cathode. In ZABs, zinc operates as the anode, oxygen from the air is an active material which experiences chemical reduction as the battery discharges once the tab from the terminal is removed, and potassium hydroxide constitutes the alkaline electrolyte. The physical cathode, termed the air cathode, is composed of carbon, additives, manganese oxide as the catalyst, and an outer polytetrafluoroethylene (PTFE) waterproof layer.

It is worth noting here that the density, quality, and grade of the materials selected for these ingredients tend to govern their output regarding energy and performance. Battery regulations refer to the ZAB as the “P” or “Primary” system classification and combine this with its “Round” or “R” shape, forming the “PR” system. It is important to note here that battery sizes will henceforth be referred to by their International Electrotechnical Commission (IEC) nomenclature, where PR48 refers to size 13 and PR44 refers to size 675. The manufacturers chosen have been anonymised to X, Y, and Z to preserve focus on the importance of battery testing.

The IEC delineate international standards for electrical and electronic devices. This provides scope for the design, fabrication, certification, and maintenance of such products. This arrangement perdures in 80% of European electrical and electronic standards, whereby regional and national laws distinctly refer to IEC standards. There are three such standards which are applicable to ZABs: IEC 60086-1:2021 Primary batteries-General; IEC 60086-2:2021 Primary batteries-Physical and electrical specifications; IEC 60086-5:2021 Primary batteries-Safety of batteries with aqueous electrolyte [2,3,4].

The International Standards Organisation (ISO) promulgate standards concerning manufacturing and technology, and at present, enact standards through 165 national standards bodies. Of the three brands, only X cites ISO standards: ISO 9001:2008 Quality Management Systems [5] and ISO 14001:2004 Environmental Management Systems [6]. The former places focus on meeting consumer expectations of performance quality, while the latter highlights the environmental footprint of the product. While these standards are commercially weighted and are appealing to business practice, the ISO also declare that these standards are still general in nature and contain ambiguous vocabulary to grant applicability to a wider base of manufacturers. Naturally, it could be argued that these standards do not offer much more than assurance of the expectations that consumers are informed they should expect from the product. This gives the manufacturer inflated authority to set the specifications to what they can or intend to supply according to the tests they select to perform. Furthermore, X’s commitment to these standards have been verified by the organisation which is certified by the British Standards Institute. Despite this declaration of conformity, these particular standards have been withdrawn by the ISO as they are outdated by their more recent, revised publications, ISO9001:2015 [7] and ISO14001:2015 [8]. X employ Statistical Process Controls (SPCs) to verify the consistency and quality of the batteries. SPCs wield statistical techniques to command the behaviour of battery testing. These are achieved through tools such as control charts to discern between the naturally occurring common cause variation, which is accepted for distribution, and the externally provoked special cause variation, which is investigated. Through an informal interview with a representative of X, this was explained to eradicate dimensional variations, where expansion of battery size risks damaging the hearing aid. While tests regarding temperature, humidity, and thermo-cycle extremes are also detailed on their website, the content of the 262 quality checks performed in manufacturing were deemed proprietary to the company.

The European Hearing Instrument Manufacturers Association (EHIMA) aims to monitor the hearing devices industry and promote the production of valid and reliable devices through recommending the uniformity of measuring regulations. The Technical Committee engages with standards authorities to assist with composing the documents, and the Regulation Committee appraises drafted regulations from the hearing industry.

While Aveyard (2014) [9] purports that studies which are three to four years old are outdated, an article by Penteado and Bento (2013) [10] was used as a frame of reference for this investigation. This was the only online study which tested zinc–air hearing aid battery (ZAHAB) performances and compared these with RHAs. A total of 10 ZAHAB brands were compared through laboratory testing of consumption, with the aim of demonstrating the most cost-effective performer available to audiologists and consumers in Brazil. The study employed IEC test conditions and discovered a significant contrast between their results and the data reported by the manufacturers.

When applied to HAs, the battery drain is dependent on the individual application of the user. Idiosyncrasies such as preferences and time spent on a particular volume level, wireless features, or programmes, in conjunction with overall daily use and levels of sound input, all impose different loads on the battery. This means a quantitative standard would have little external validity. However, IEC 60086-1:2021 [2] delineates how battery discharge should be assessed, and records two approaches of doing so; Application testing and Service Output testing. The former may be used to analyse whether the batteries being tested are best suited for application in a HA. The latter may reveal the overall effectiveness of this application through time. A restricted length of 30 days is also suggested for testing the battery discharge.

The Application test employs the operating end-point voltage (EPV), current load, and equivalent resistance load from typical application use. While the former two are obtained through measurement, the equivalent resistance load is calculated from the average current and average operating voltage under load. The median class average is employed to define the load value and EPV value used for calculating the discharge. The standard proceeds to note the considerations for this test whereby typical duty cycles and the efficiency of discharge load through the application are influential factors. The standard also notes the limitation of how patterns of usage can mutate the longevity of battery life through conditions which may be favourable or unfavourable, such as the device’s demand of current; the frequency of current demand (continuous or intermittent usage); the minimum voltage at which the device will satisfactorily operate (EPV); and the temperature of operation. Furthermore, the standard suggests that, in the future, improved load conditions will be implemented to more accurately reflect the applications which are developing for this technology.

This declaration appears to acknowledge the difficulty of composing standardised tests. As multiple loads are key to resembling real-life HA use, the standard advises that a cycle should begin with the heaviest load and transition to the lighter load. An objective pattern analysis of data logging 15,905 HA users by Pasta et al. (2021) [11] found that, over four months, the average daily use was 10.01 h. The within-user and between-user variability were found to be significant: 2.76 h and 3.88 h, respectively. However, the study procured the data from participants through a smartphone app which connects to their HAs. This may inherently skew the demographic to users which have more confidence in managing electronic devices such as HAs, in addition to those who have access to smartphone and wireless aid technology. Yet, the participants may be more reflective of future HA users, as acclimatisation to technology in earlier life and the implementation of HAs which offer new features are phenomena which are being observed in HA users at present (Karawani et al., 2022 [12]; Maidment and Amlani, 2020 [13]). The participant’s experience of battery consumption during engagement with wireless features and general use were not discussed in the study; therefore, it is difficult to compare hourly discharge periods with IEC, EHIMA, and manufacturer data. However, participants were clustered according to their HA use, which may be a more accurate and personalised method of measuring and predicting battery consumption. Comparatively, a study by Arthur et al. (2017) [14] analysed the HA use of 119 patients in Wales through comparing data logging with self-reported Glasgow Hearing Aid Benefit Profiles. Contrastingly, the study found the average use was 5.87 h a day; however, users who reported 100% use produced an average of 9.94 h a day. The study concludes by noting the importance of lifestyles and correlating needs; for example, a user in a quiet office will likely have different demands to a user working in a busy public space. McCormack and Fortnum (2013) [15] posited that complex psychosocial issues, device stigma, health professional’s attitudes towards devices, and the perceived value of HAs are factors contributing to low or non-use. Correspondingly, Williger and Lang (2015) [16] discovered that satisfaction with and the effectiveness of HA devices influence low or non-use.

It would perhaps be prudent to ask why ZAHABs are not screened through an application test, as opposed to a service output test, enabling the reliability of their performance in the HA to be validated. Despite the idiosyncrasy of usage patterns, a catalogue of such patterns with a correlating time frame may be beneficial to both audiologists and patients for performance expectations and troubleshooting. Zhao et al. (2019) [17] note the shortfalls of ZABs and propose directions for further study, including the process of passivation during discharge, where the zinc oxide forms an irreversible passivation layer mainly on the anode, increasing the internal resistance of the battery (Liu et al., 2024) [18].

The EHIMA recommendations are similar to the discharge testing described in the IEC standards. EHIMA notes that these conditions stray from the IEC standards, yet do not explain why. Naturally, there is a growing expectation of battery life in correlation to the increasing battery size and the significance of load on battery performance. However, the justification for each load is not given, and so it is difficult to accurately interpret the meaning of the pulse and background loads. It is worth questioning what these loads are representing, as HA features or environmental settings which demand a higher current to last for 100 ms is conceivably unrealistic. On the contrary, it may also be difficult for HA users to replicate a constant low-level sound environment over extensive time periods. Evidence of studies substantiating these values would be beneficial for manufacturers to understand and develop their own values for consensus among committees. Although IEC 60086-1:2021 acknowledges the impracticality of setting numerical values for battery capacity standards, the document also acknowledges the imperative of refining consumer expectations. However, there appears to be a mismatch between the test types and the test conditions; for example, EHIMA’s high power drain proposes the same test conditions as IEC’s *standard drain*. However, as there is such a contrast, it may be within reason to suspect that EHIMA and the manufacturer’s conditions refer to the optimal extension of battery life, which counters the IEC’s minimum duration.

The scope of this study is well suited to translational research in audiology, as it places focus on the functional elements which influence hearing aid performance and thus the users’ benefit, allowing questions to be posed which cannot be answered in clinics. Due to the deficit of knowledge discovered in this field of applied audiology, this research may also indicate discrepancies between test parameters which may have weighting to clinical practice and patient use. Therefore, an investigation into the performance of ZAHABs would inform consumer expectations, as the technology behind hearing aid function plays a fundamental role in patient experience and offers an improved insight into the requirements of battery testing, leading to enhanced consumer choice through informed decision-making. The following hypotheses were constructed from this study of the literature:

**H_0_:**  *There is no significant difference between manufacturer and standard battery service hours.*

**H_1_:**  *Brand Z will demonstrate the longest service hours, but no manufacturer will perform to expected service hours. This is based on previous evidence (Penteado and Bento, 2013).*

## 2. Methods

### 2.1. Literature Search Strategy

An exploratory search involved grey literature due to the lack of peer-reviewed research in this field. This was achieved following Preferred Reporting Items for Systematic reviews and Meta-Analyses (PRISMA) Checklist 2020 [19], using the search engine “Google” and electronic databases such as NCBI, PubMed, IEC Standards, and Swansea University’s “ifind”. This involved the terms “hearing aid battery”, “zinc-air battery”, “zinc-air cell”, “zinc-air button”, “standards”, “specification”, and “laws” in combination. However, due to the heterogeneity in the applications of ZABs, studies not devoted to primary batteries was excluded from the search. Boolean phrases were oriented to dilute and refine searches. The terms “battery”, “button”, and “cell” are used interchangeably in the description of the ZAHABs because they meet all definition criteria in Section C.2.4 of Annex C of IEC 60086-1:2021 [2].

### 2.2. Laboratory Experiment on ZAHAB Service Output

Due to the financially and temporally restrictive elements of the study, only four cells of each PR48 and five cells of each PR44 battery per manufacturer were tested. Y and Z batteries were acquired in batches from the online marketplace *Amazon*, while the X batteries were procured from the company. All batteries were selected as they had the same expiry date.

A consultant medical engineering company was recruited to perform the experiment due to their specialist knowledge and equipment. For the battery holder, a coin cell holder was soldered onto a Veroboard, ensuring the positive and negative terminals of each holder were not connected, and that there were no connections between separate battery holders if more than one was fitted to the same Veroboard. Rubber feet were fitted under the Veroboard to further isolate the battery holder terminal from any other noise source. A Keysight Technologies DC Power Analyser recorded voltage, current, and time measurements from each battery terminal, and was programmed in a controlled laboratory setting to impose cycled loads on the battery cells. Although the IEC recommends that eight cells be tested during sampling, only five of PR44 and four of PR48 were used due to the restricted time frame of this project. These loads followed EHIMA High Power (HP) test conditions, as the IEC and manufacturers also adopted these conditions, and thus enabled direct comparison of results between these sources. The standard (STD) and HP test conditions are detailed in EHIMA Recommendations for Zinc-Air Hearing Aid Batteries Version 2.0 [20]. Each battery was tested daily in six repeated cycles, beginning with pulse load for 100 ms, followed by the background load for 119 min, 59 s, and 900 ms. After these load periods, the cycle is completed with an off period of 12 h, and then repeated from the beginning. When the EPV reached 1.10 V, in either background or pulse load, this signalled the conclusion of service life. A comparison was made based on their voltage drop over these cycle periods. It is important to note that the first discharge cycle is started no less than one minute after the battery’s activation.

For the standard (STD) application test of the PR48 type, the background current was set to 2 mA and pulse current was set to 6 mA. For HP test conditions, the background current was set to 3 mA, and the pulse current was set to 12 mA.

For the standard application test of the PR44 type, the background current was set to 5 mA, and the pulse current was set to 15 mA. For HP test conditions, the background current was set to 8 mA, and the pulse current was set to 24 mA.

The DC Power Analyser was designed so that the Output Source Settings configured each channel to emulate (at 2 Quadrant Power Supply) and operate at the current priority. An arbitrary waveform was created where the arbitrary type was current and output type was sequence. The waveform was set to match the discharge conditions described in the HP tests above.

Table 1 and Table 2 below catalogue the equipment used and the method of data processing, respectively. Table A1 (Appendix A) illustrates the EHIMA discharge conditions in greater detail.

These test conditions ensured the same cell size for each manufacturer was only tested once on the same channel. This reduced any possible undetected bias potentially introduced by a particular channel. The equipment was calibrated to the standard (see Appendix A). The temperature was maintained at 23 °C ± 0.5 °C and relative humidity at 50 ± 25%; however, a further test at room temperature may have accelerated consumption. IEC recommends a temperature of 20 ± 2 °C and relative humidity of 55 ± 10% RH for discharge testing. The power cuts which occurred on certain test runs caused millisecond-period interruptions and may have influenced the final results; however, these were re-run on a second channel to check for disturbances.

The software IBM Statistical Package for the Social Sciences (SPSS) version 26 was implemented for the One-way Analysis of Variance (ANOVA) test for statistical significance between the results.

## 3. Results

**Figure A1:**
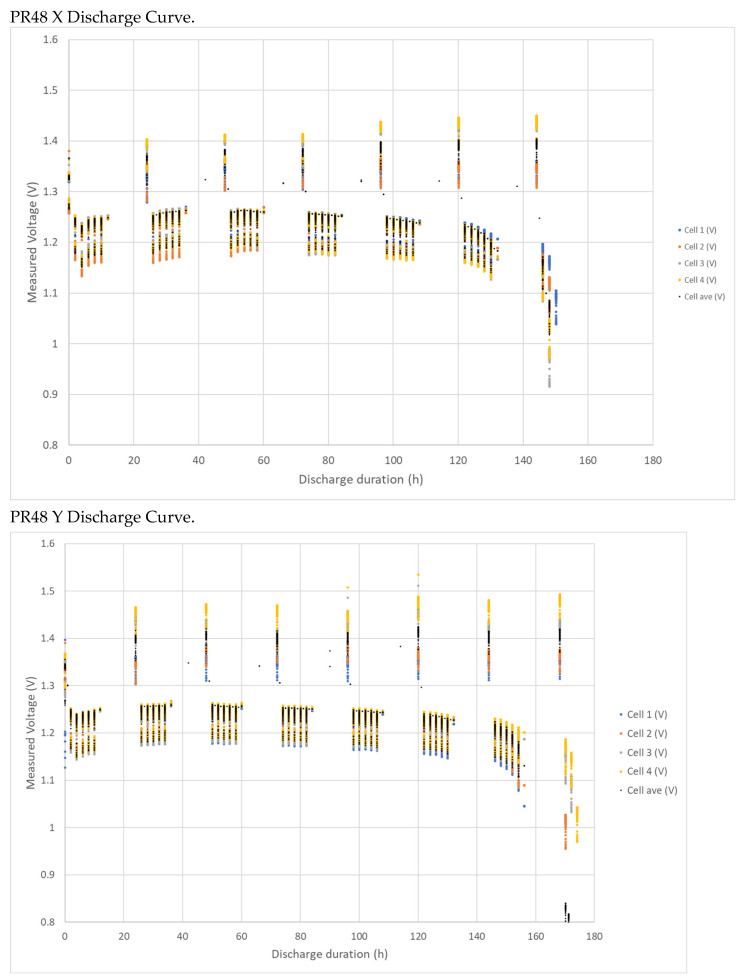

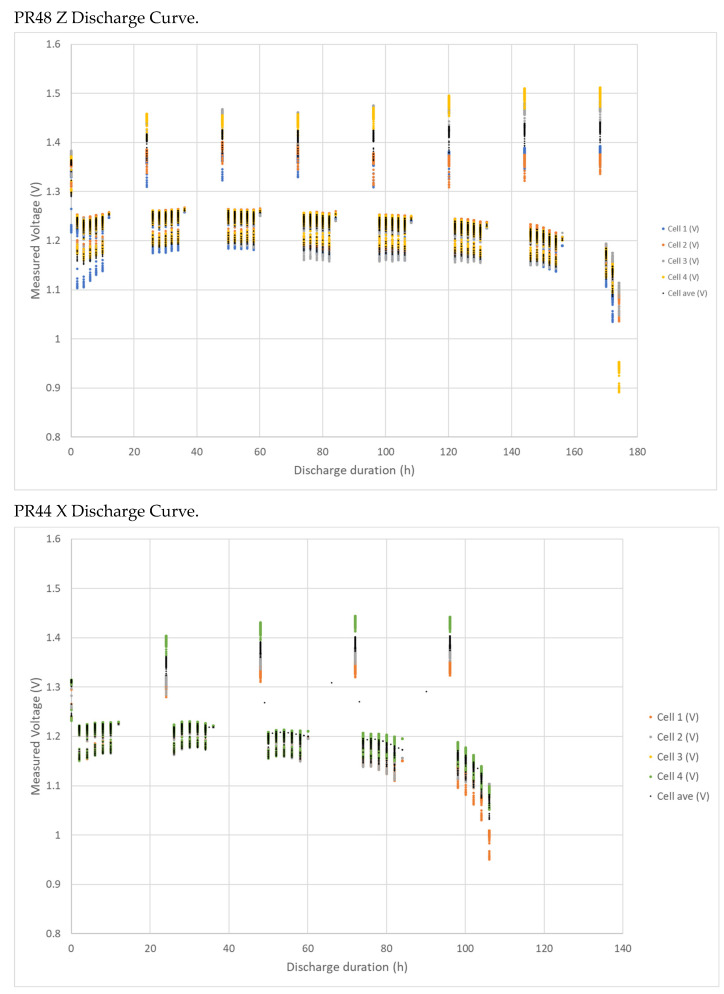

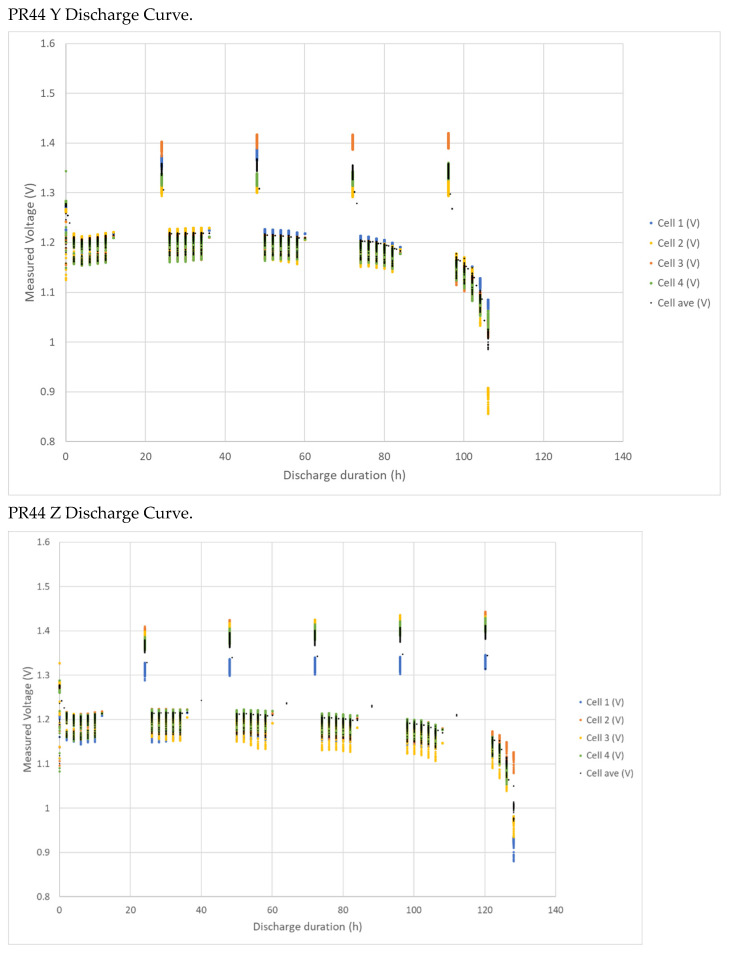
S1: PR48 and PR44 averaged discharge curves for X, Y, and Z manufacturers.

The averaged results are shown in Table 3, rounded to three decimal places. Table A2 and Table A3 in Appendix B provide the raw data collected from the individual cells. Appendix C (Figure A1) is a collation of the graphical illustrations of the data.

A One-way ANOVA confirmed that the null hypothesis should be rejected, as the battery service hours were significantly different between the three PR44 manufacturers (F(2,12) = 8.479, *p* = 0.00506). Likewise, the same analysis for the PR48 battery manufacturers revealed significantly different service hours, (F(2,9) = 40.438, *p* = 0.00003), so the null hypothesis for this battery type can also be rejected.

## 4. Discussion

The results confirm the alternative hypothesis (H_1_) that brand Z ZAHABs appeared to outperform X and Y in both the PR48 and PR44 sizes. However, further analysis is needed to test whether this is a statistical difference or the under or over performance by one or more of the brands. The null hypothesis (H_0_) can therefore be rejected. Table 4 examines adherence to manufacturer, IEC, and EHIMA standards.

In alignment with H_1_, Table 4 indicates that all three brands meet the IEC standard hours of minimum average duration, yet all underperform in comparison to their manufacturer’s reported service hours. Both Z and Y’s PR48 batteries perform to the EHIMA’s recommendations, whereas Y’s PR44 battery and both of X’s batteries fall short of the EHIMA recommended service hours.

Whilst the exploration of the literature concluded that background and pulse test conditions were unaligned with realistic use, the HP consumption may help to balance this slightly as it is more aligned with the results from Pasta et al. (2021) [11] and Arthur et al. (2017) [14]. HP conditions also enable a potential representation of acoustic feedback, volume control, and the use of programmes available in HAs, through the logic that greater amplification draws more current which increases battery consumption. The method described by Penteado and Bento (2013) [10] was not adopted because it did not use pulse and background loads, which potentially erodes the validity of comparing it with manufacturer and IEC data. The method patented by the Timex Group USA Inc. (1998) [21] was also not chosen due to its long duration and extensive use of equipment. Section 7 of IEC 60086-1:2021 [2] stipulates that, where no agreement between tester and manufacturer has been made, ISO 2859 [22] and ISO 22514-2 [23] should be referred to for guidance on sampling and quality compliance assessment.

The study by Penteado and Bento (2013) [10] could not identify a superior brand due to certain sizes from different brands performing better; yet, out of X, Y, and Z, the latter produced the longest service life in both the PR48 and PR44 sizes. However, it should be noted that a test jig was constructed to collect the data, and there is no mention of calibration or reliability of the circuit. A potential weakness of this study was the ambiguity of the battery lines tested, where they do not specify which product models were chosen and thus there is little possibility of any comparisons being made with the present study. Furthermore, the graphical data provided are published with a low-resolution, meaning interpretations of the figures are difficult to assert. The study proceeds to compare the average service capacity with a rechargeable hearing aid (RHA) battery produced by the company Solar Ear. The study found that the average service hours of PR44 were five times greater and PR48 were seven times greater than their rechargeable counterparts. However, Solar Ear did not provide test conditions for their product, and the process of charging the HA batteries required #AA batteries to be charged initially, meaning the service performance is somewhat shared with the brand of #AA batteries used. Furthermore, as the only rechargeable brand tested, this may not be externally valid for all RHA technology. On balance, this study delivers crucial insights into the methodology of testing HA batteries, and as the only study found to investigate this area, was therefore chosen a frame of reference for the experiment.

Observing both ZAHAB sizes, it can be seen that the X battery underperformed its reported service life by an average of 10.5 h, Y underperformed by an average of seven hours, and Z underperformed by an average of 97.5 h. It is unclear why Z set their reported service life as exceedingly different from the other manufacturers, as it is assumed that such significant differences in technology are difficult to yield.

Combining the average daily use found in Pasta et al. (2021) [11] and Arthur et al. (2017) [14], discussed in the literature review, an average of 9.98 h was calculated. This value was then applied to calculate how many days of use an HA user would receive from the average service life of each manufacturer’s battery under HP conditions, respectively. For the PR48 battery, we calculated 7.5 days, 8.5 days, and 8.9 days. For the PR44 battery, we calculated 5.2 days, 5.3 days, and 6.3 days. These estimations indicate that the HP test conditions appear to be relevant to modern HA user patterns and expectations, which consequently challenges the relevance of the standard drain conditions. These expectations may be a valuable groundwork for improving patient insight, with the adoption of terms such as light, moderate, and heavy use to explain the correlation between patient usage patterns and estimated hours of service life. However, further research would be required to firmly establish correlations between capacity profiles and behaviour patterns.

To investigate why performance variations have occurred would require a greater examination of the factors affecting battery performance. These are pertinent to the quality assessments carried out by manufacturers and ensure suitable storage conditions for shipments, Audiology departments, and hearing aid users. The online literature [24,25,26] has been synthesised to provide insight into how users can avoid premature depletion: the user’s hearing loss; greater amplification is required to rehabilitate greater degrees of hearing loss, which increases the current drawn and shortens the battery life. This logic also applies to the specific hearing aid model, where more powerful aids are prescribed to more severe hearing losses, according to the ability of the user to programme the aid, the receiver chosen, and user preferences such as increasing the amplification through the volume control on the aid for any length of time. Feedback refers to a fault within the aid, where sound is processed excessively, meaning more current is drawn from the battery unnecessarily. External acoustic feedback is caused by an ill-fitting earpiece, or providing too much high frequency gain outside the recommended boundaries of the hearing aid. Internal acoustic feedback is caused by sound leakage within the hearing aid itself. Mechanical feedback occurs when physical vibrations occur due to the contact of the receiver with some aspect of the hearing aid casing. Electromagnetic feedback occurs when the hearing aid is set to the telecoil loop setting, where this radiation is emitted by the receiver and picked up by the telecoil and re-amplified. Sound Environment: usage patterns such as the reception and processing of elevated or constant sounds will draw more current and reduce the battery life. A battery will last longer in a library than in a restaurant. Humidity: too much or little water in the air can either dry out the battery or cause it to absorb more moisture, which interferes with the natural discharge expansion. Both will reduce battery life. The desirable relative humidity is 55 ± 10% RH. Temperature: lower temperatures decrease the voltage, which limits the amount of current which can be drawn, and thus shortens the battery life. The desirable storage temperature is 20 ± 2 °C. Altitude: with greater altitudes, there is a reduction in the percentage of oxygen in the air. As the battery relies on oxygen as the cathode, less reactions can take place, causing the end-point voltage to be met sooner, meaning a restricted battery life. Magnetic Fields: if stored proximally to technologies which also produce magnetic fields, such as computers and phones, the battery function will be distorted, thus reducing its life. Hearing aid technology: the more advanced the aid is, the more power is required. Modern technological features like noise cancellation and multi-channel processing can reduce battery life by 20%. FM looping, sound generation for tinnitus patients, and wireless or Bluetooth can increase the current by up to 300%, which shortens battery life significantly. Current demands (mAh) on hearing aid batteries depend on the features used: streaming the radio uses 4.32 mA; streaming from a microphone uses 4.28 mA; a Bluetooth phone uses 4.27 mA; wireless programming uses 3.17 mA; and the average hearing aid uses 1.94 mA. Battery Materials and Handling: handling the battery with unclean fingers may cause plaques to form through dirt or oils, which reduces the surface area of the zinc anode, meaning fewer reactions with oxygen are able to take place through ionic resistance. In turn, the voltage is restricted, which reduces the potential life of the battery. High quality, grade, and density materials improve battery performance and service life.

Antithetically, one should also evaluate the limitations of the data collected and adopted methodology; the batteries were purchased from an online marketplace and so do not disclose the conditions that the batteries were stored in. It is important to note that IEC advises the testing of eight batteries; however, due to the time scale of the project, only four PR48 and five PR44 batteries were tested per manufacturer. In addition to this, the IEC reports that a high current demand for prolonged periods coupled with a high cut off voltage and low temperature represents the worst-case conditions resulting in significant capacity loss. Other limitations reside in the analysis and presentation of the data, as one-way analysis of variance relies on assumptions such as sample independence and variance equality, and averaged data may indicate an oversimplification and are sensitive to anomalous results.

It would be of interest to hearing aid users to report and compare the longevity of their ZAHABs in correlation to their hours of use. From anecdotal evidence from audiology clinics, patients have shown an awareness of this variability in longevity, and yet there is no supporting evidence which clinicians can cite to advise the patient with. Therefore, this study may provide a form of evidence for clinicians to reference when discussing differences in battery performance. However, it is evident that further analyses into the performance of different battery types for each manufacturer, in conjunction with hearing aid manufacturers, should be explored. A service evaluation of patient reported battery performance, using the data logging of the hours of use in hearing aids, may be useful here. Moreover, it may also be valuable for the IEC to adjust their policy to provide greater transparency regarding deviations in longevity, and perhaps even reconsider the suitability of Application tests for standard and high-power drains. It should be recognised that the short, high current pulses of 100 mS employed in EHIMA and IEC test conditions are not realistic for real world HA operation. Furthermore, these sudden pulses of high current are likely to exacerbate the issue of zinc electrode passivation by ZnO, thus reducing the overall discharge capacity of the battery. Therefore, a testing regime of gradually changing current loads may result in more accurate reflections of real-world performance, and possibly improved battery life.

## 5. Conclusions

On balance, the experiment demonstrated the significance of intra- and inter-variability in battery performance, and in agreement with Penteado and Bento (2013), generally reflects the idiosyncratic patterns of use by HA users. As HP test conditions provided the service life expected from standard drain test conditions, the modern-day relevance of the latter should be assessed. This lack of consistency may be useful for audiologists to note when fitting HAs or discussing batteries with patients. Overall, Z’s battery provided the longest service life. This study highlights how standardised battery tests are unrepresentative of real-world use, where significant variations in discharge capacities have indicated a need to advance research into hearing aid usage patterns, and for these to be involved in battery testing regimes.

## Figures and Tables

**Table 1:** Equipment used in battery performance tests.

Equipment Description	Calibration Due Date
Electronics laboratory capable of maintaining 23 °C ± 0.5 °C and 50 ± 25%RH	N/A
Tutela temperature probe for laboratory 2G7	3 August 2022
Tutela humidity probe for chamber 2G7	3 August 2022
Keysight Technologies DC Power Analyser	5 January 2023
Keysight Technologies DC Power Analyser	10 January 2023

**Table 2:** Methods used in data processing of battery performance test.

Method Description	Data Processing
	1. .dlog files: logs captured by DC power analysers, containing voltage and current measurements sample once every 12.5 ms (8 samples per 100 ms). Readable by Keysight 14585A software. 2. .csv files: logs exported to .csv format. 3. _parsed.csv files: data extracted from .csv files using the following criteria: 4. Voltage and current readings extracted from start of log until first pulse current application (gives information on unloaded activation period voltages) 5. Voltage and current readings extracted for each pulse current application thereafter, and ~10 sample of background current application before and after each pulse current application 6. Results template: _parsed.csv files processed to calculate the *Service Duration* (h) and *Rated Capacity* (mAh) achieved by each cell.

**Table 3:** ZAHAB average service performance results.

Size	Manufacturer	Average Service (Hours)	Average Service Capacity (mAh)
PR48	X	75.770	227.309
	Y	84.780	254.341
	Z	89.289	266.869
PR44	X	52.247	417.977
	Y	52.890	423.122
	Z	63.306	506.449

**Table 4:** Comparison of results with manufacturer, IEC, and EHIMA values.

Size	Battery Brand	Average Service (Hours) from Present Study (3.d.p)	Manufacturer Reported Service (Hours)	IEC Standard (Minimum Average Duration of Hours)	EHIMA Recommended Service (Hours)
PR48	X	75.770	86	55	80
	Y	84.780	90	55	80
	Z	88.956	196	55	80
PR44	X	52.247	57	45	60
	Y	52.890	60	45	60
	Z	63.306	150	45	60

## Data Availability

The original data presented in the study are openly available in FigShare at https://doi.org/10.6084/m9.figshare.25273480.v1.

## Appendix A

**Table A1:** Implementation of EHIMA discharge conditions.

Step	Function Type	Time (s)	Current (A)
0	Step	t_0_ = 600	I_0_ = 0
		t_1_ = 0.1	I_1_ = Pulse Current
1	Pulse	t_0_ = 7199.9	I_0_ = Background Current
		t_1_ = 0.1	I_1_ = Pulse Current
		t_2_ = 3599.95	I_2_ = Background Current
2	Pulse	t_0_ = 3599.95	I_0_ = Background Current
		t_1_ = 0.1	I_1_ = Pulse Current
		t_2_ = 3599.95	I_2_ = Background Current
3	Pulse	t_0_ = 3599.95	I_0_ = Background Current
		t_1_ = 0.1	I_1_ = Pulse Current
		t_2_ = 3599.95	I_2_ = Background Current
4	Pulse	t_0_ = 3599.95	I_0_ = Background Current
		t_1_ = 0.1	I_1_ = Pulse Current
		t_2_ = 3599.95	I_2_ = Background Current
5	Pulse	t_0_ = 3599.95	I_0_ = Background Current
		t_1_ = 0.1	I_1_ = Pulse Current
		t_2_ = 3599.95	I_2_ = Background Current
6	Step	t_0_ = 3599.95	I_0_ = Background Current
		t_1_ = 10,000	I_1_ = 0
7	User	t = 10,000	I = 0
8	User	t = 10,000	I = 0
9	User	t = 10,000	I = 0
10	User	t = 3200	I = 0

## Appendix B

**Table A2:** Individual cell data of PR44 battery performances.

PR44 Service Duration (h)									
	Y			Z			X		
	Test Start Date	Service Duration (h)	Service Capacity (mAh)	Test Start Date	Service Duration (h)	Service Capacity (mAh)	Test Start Date	Service Duration (h)	Service Capacity (mAh)
Cell 1	3 August 2022	82.271	246.814	18 August 2022	88.288	264.864	11 August 2022	78.270	234.809
Cell 2	3 August 2022	82.271	246.814	18 August 2022	90.288	270.864	11 August 2022	76.270	228.809
Cell 3	3 August 2022	86.289	258.868	18 August 2022	90.290	270.871	11 August 2022	74.269	222.808
Cell 4	3 August 2022	88.289	264.868	18 August 2022	88.290	264.871	11 August 2022	74.269	222.808
Minimum		82.271	246.814		88.288	264.864		74.269	222.808
Average		84.780	254.341		89.289	267.868		75.770	227.309

**Table A3:** Individual cell data of PR48 battery performances.

PR48 Service Duration (h)									
	Y			Z			X		
	Test Start Date	Service Duration (h)	Service Capacity (mAh)	Test Start Date	Service Duration (h)	Service Capacity (mAh)	Test Start Date	Service Duration (h)	Service Capacity (mAh)
Cell 1	13 May 2022	56.245	449.962	13 May 2022	66.262	530.098	27 May 2022	44.235	353.883
Cell 2	27 May 2022	45.469	363.752	27 May 2022	55.486	443.887	24 June 2022	50.240	401.919
Cell 3	1 July 2022	54.254	434.032	1 July 2022	68.264	546.110	24 June 2022	54.240	433.919
Cell 4	13 July 2022	54.238	433.905	1 July 2022	62.264	498.110	19 July 2022	56.260	450.081
Cell 5	19 July 2022	54.245	433.961	13 July 2022	64.255	514.040	19 July 2022	56.260	450.081
Minimum		45.469	363.752		55.486	443.887		44.235	353.883
Average		52.890	423.122		63.306	506.449		52.247	417.977
